# Numerical Modelling of the Optical Properties of Plasmonic and Latex Nanoparticles to Improve the Detection Limit of Immuno-Turbidimetric Assays

**DOI:** 10.3390/nano11051147

**Published:** 2021-04-28

**Authors:** Giuliano Coletta, Vincenzo Amendola

**Affiliations:** Department of Chemical Sciences, University of Padova, Via Marzolo 1, 35131 Padova, Italy; giulianocoletta1994@gmail.com

**Keywords:** gold nanoparticles, silver nanoparticles, latex nanoparticles, plasmon, optical properties, turbidimetry, DDA

## Abstract

Turbidimetric assays with latex nanoparticles are widely applied for the detection of biological analytes, because of their rapidity, low cost, reproducibility, and automatization. However, the detection limit can be lowered only at the price of a reduced dynamic range, due to the rapid saturation of the light scattering signal at high analyte concentration. Here, we use numerical calculations to investigate the possibility of increasing the performance of immuno-turbidimetric assays without compromising the measurement dynamic range, by combining plasmonic (gold, silver) and latex nanoparticles. Our modelling results show that plasmonic nanoparticles are compatible with a large signal change even when small aggregates are formed, i.e., at low analyte concentration. The working principle relies on the remarkable modification of the surface plasmon band when noble metal nanoparticles form oligomers, and also when latex particles are included in the aggregate. At high analyte concentration, when larger aggregates form, the latex particles can provide the required linear response of standard immuno-turbidimetric assays. Thus, the combination of the two components can be a successful strategy to improve the detection limit and the dynamic range, while maintaining all the advantages of the homogeneous immuno-turbidimetric assays.

## 1. Introduction

In turbidimetric assays, the quantity of the analyte is correlated to optical extinction through a colloid of nanoparticles (NPs) that selectively undergo aggregation and scattering of light in the presence of the target compound [1,2,3,4]. These assays are widely used in medicine for measuring the concentration of various biological components but find application also in environmental, food, and agriculture analysis [3,5,6]. Immuno-turbidimetric assays usually rely on surface functionalized submicrometric latex spheres because of their low cost, uniform tailorable diameter, ease of surface functionalization with selective targeting units, colloidal stability, and linearity of the light scattering intensity in the visible range at increasing levels of aggregation [1,2,3,4,6,7]. The intensity of the light scattered by these particles exceeds the luminosity of an organic fluorescent molecule by several orders of magnitude [4,8]. The latex spheres are functionalized with antibodies or other molecular functions capable of selective binding of the analyte in a sandwich configuration, which implies a growing aggregation with increasing analyte concentration, and a consequent growing light scattering intensity [1,2,7,9]. The resulting immuno-turbidimetric assays gather together a remarkable range of positive features: they are rapid, phase homogeneous, washing-free, single-step, low cost, selective, reproducible, automatable, and, so far, are among the standard methods for detection of bioanalytes of clinical interest, in particular proteins that are well recognized by antibodies [1,2,6,7].

The detection limit of immuno-turbidimetric assays can be lowered by increasing the size of the latex nanospheres because the light scattering signal and its variation upon nanosphere aggregation both scale with the 6th power of particles radius [2,6,10,11,12,13]. For the same reason, however, the large latex particles introduce a high background signal in turbidimetric assays [4,6,14], which rapidly saturates beyond the instrumental photometric range at high analyte concentration, forcing sample dilution when a quantitative measurement is required [1,2]. Lowering the latex concentration is not a good strategy, because this is accompanied by the growth of the detection limit and the saturation of the available targeting units on the nanospheres with consequent saturation of the response, even if the total signal is within the spectrophotometric range of the turbidimeter (i.e., an optical absorption spectrometer working at a specific wavelength interval) [2,6]. Nonetheless, there is a continuous demand for lowering the detection limit to allow the use of small volumes of samples, such as in microfluidic circuits [7,15].

An approach to solve this problem is the combination of latex spheres of different sizes (127 and 220 nm) and functionalized with targeting molecules (for instance, antibodies) with different level of affinity for the analyte [1,2]. When the large (220 nm) latex particles have a high affinity with the analyte, these will provide high sensitivity (i.e., change of signal for a unit change of the quantity of analyte) at low analyte concentration. When analyte concentration increases, the population of 220 nm latex spheres is saturated by the analyte before reaching signal saturation in the instrument, whereas the small (127 nm) latex spheres bearing the antibodies with lower affinity are still available to react, providing a growing signal in the concentration range where sensitivity is not an issue.

Although this principle is successful in most cases, tailoring the exact proportion of large and small nanospheres and selecting the appropriate combination of targeting molecules with different affinity to meet the demanded dynamic range and detection limit is not an easy task with analyte varying over several orders of magnitude of concentration. This may often result in unsatisfactory linearity in the whole measurement range. Moreover, large latex nanospheres may pose problems of reproducibility during the analysis due to aspecific agglomeration [4,6] or may affect the long-term storage of the reagent, which usually should be guaranteed for at least one year [6,14]. A more subtle problem is observed with large nanospheres, because of their large volume compared to the analyte, and consequent inertia in the formation of immuno-aggregates at very low analyte concentration.

Here, we investigated a different approach that is the combination of plasmonic noble metal NPs (Au and Ag) with latex nanospheres to achieve a low detection limit, high sensitivity, and large dynamic range at the same time. Au and Ag NPs have intense extinction bands in the visible range due to the collective excitation of conduction electrons (plasmons) [14,16,17,18]. These NPs have been widely exploited for spectrophotometric detection of analytes reaching a very low detection threshold also in homogeneous assays where the nanoparticles are mixed directly with the analyte in the liquid phase instead of being supported on a substrate [14,15,18,19,20]. Although in the subwavelength scale, advanced optical structures like self-assembled plasmonic nanoarrays and metamaterials provide significant sensitivity in biosensing assays, leading to the emergence of biosensors with an ultralow limit of detection at very low analyte concentrations [21,22,23], the homogeneous assays still have the advantage of low-cost and high-throughput in flow continuous analysis without the need for continuous replacement of the detection substrate for each sample [24,25,26].

The success of Au and Ag NPs in biosensing is connected to the large extinction (i.e., absorption plus scattering [16,17]) cross-section at the plasmon resonance band and to the rapid increase of optical density at longer wavelengths upon particles aggregation [14,15]. The appearance of plasmon resonance bands in the red and near-infrared is due to the electric dipole–dipole interaction and coupling between the plasmonic modes of neighbouring particles in the cluster [14,17,18,27,28]. However, the dynamic range of the assays based on agglomeration of plasmonic NPs is also limited by the nonlinear increase of plasmon extinction versus the number of particles in the aggregate, which typically follows a sigmoidal trend versus the logarithm of the analyte, with a tight linearity interval not suited for the assay of species whose concentration changes over several orders of magnitude [18,29,30]. This is mostly due to the continuous red-shift of the plasmon band while increasing the aggregate size [14,17,18,27] that is not compatible with standard turbidimetric detection at a fixed wavelength.

Here, we focused on a different approach, combining the high sensitivity of the plasmon resonance in noble metal NPs with the excellent linearity of latex spheres with a size of the order of 100 nm. More specifically, we systematically modelled the change in the optical properties when NPs go from the monomeric to the oligomeric (dimer, trimer, etc.) state, as a function of particles composition (latex, Au, Ag, and their combination) and size. A variety of interesting optical effects are possible in hetero-aggregates of plasmonic and dielectric nanoparticles, including the appearance of magnetic plasmonic resonances and dark plasmonic modes or light localization phenomena [17,31,32].

The optical response of oligomers is the most relevant when pursuing high sensitivity of immunoturbidimetric methods at low analyte concentration and where small latex spheres fail [1,2,33]. At larger analyte concentration, the response of commercial latex beads is already satisfactory when the spheres have a size of ca. 100 nm, not requiring further efforts of optimization. The results of the extended modelling effort point to the use of a mixture of plasmonic and latex nanospheres as a promising strategy to obtain turbidimetric assays with a low detection limit and a wide dynamic range. 

## 2. Materials and Methods

The extinction cross-sections (*C_ext_*) of NPs with different composition, size, and arrangement in aqueous solution were evaluated with the Discrete Dipole Approximation (DDA) method [34,35]. In DDA, the structure of interest, called “target”, is modelled with a number N of polarizable points (dipoles) arranged in a cubic lattice with the same geometry and permittivity of the original object [34,36,37,38]. The basis of the DDA is that the polarization *P_j_* induced on each dipole *j* of position *r_j_* and polarizability *p_j_* is given by [34,36,37,38].

(1) $$\bar{P_{j}}=p_{j}{\bar{E}}_{Loc}\left({\bar{r}}_{j}\right)$$

where *E_Loc_* is the electric field originated by the incident radiation of amplitude *E_0_*, and includes the contribution of all other dipoles [34,36,37,38]:

(2) $${\bar{E}}_{Loc}\left({\bar{r}}_{j}\right)={\bar{E}}_{0}\exp\left(i\bar{k}\cdot {\bar{r}}_{j}+i\omega t\right)-\sum_{l\neq j}{\bar{\bar{A}}}_{jl}{\bar{P}}_{l}$$

where ${\bar{\bar{A}}}_{jl}$ is the interaction matrix and ${\bar{E}}_{inc}\left({\bar{r}}_{j}\right)={\bar{E}}_{0}\exp\left(i\bar{k}\cdot {\bar{r}}_{j}+i\omega t\right)$ is the incident monochromatic plane wave with frequency *ω* and wavevector $\bar{k}$.

The full expression of ${\bar{\bar{A}}}_{jl}{\bar{P}}_{l}$ is: [34,36,37,38]

(3) $${\bar{\bar{A}}}_{jl}{\bar{P}}_{l}=\frac{\exp\left(ikr_{jl}\right)}{r_{jl}{}^{3}}\left\{k^{2}{\bar{r}}_{jl}\times \left({\bar{r}}_{jl}\times {\bar{P}}_{l}\right)+\frac{\left(1-ikr_{ij}\right)}{r_{jl}{}^{2}}\left[r_{jl}{}^{2}{\bar{P}}_{l}-3{\bar{r}}_{jl}\left({\bar{r}}_{jl}\cdot {\bar{P}}_{l}\right)\right]\right\}$$

where ${\bar{r}}_{jl}={\bar{r}}_{j}-{\bar{r}}_{l}$, $r_{jl}=\left|r_{j}-r_{l}\right|$.

The extinction cross-section of the target is then given by [34,36,37,38]

(4) $$C_{ext}=\frac{4\pi k}{{\left|{\bar{E}}_{0}\right|}^{2}}\sum_{j=1}^{N}\left({\bar{E}}_{inc}^{*}\cdot {\bar{P}}_{j}\right)$$

where ${\bar{E}}_{inc}^{*}$ is the complex conjugate of the incident electric field. 

In this work, calculations were performed with the DDSCAT software [39], where *p_j_* is expressed according to the lattice dispersion relation (LDR) developed by Draine and Goodman [34,39], i.e., as a correction of the Clausius–Mossotti polarizability by a series expansion of *k ^.^ d* and *ε_m_*, with *d* the interdipole spacing and *ε_m_* the matrix dielectric constant [34,36,37,38]:

(5) $$p_{j}^{LDR}=\frac{p_{j}^{CM}}{1+p_{j}^{CM}\left[b_{1}+b_{2}\varepsilon_{j}+b_{3}S\varepsilon_{j}^{}\right]\left(\frac{k^{2}}{d}\right)}$$

where *ε_j_* is the dipole permittivity, *b*_1_, *b*_2_, *b*_3_, and *S* are coefficients of the expansions and $p_{j}^{CM}$ is the Clausius–Mossotti polarizability [34,36,37,38]

(6) $$p_{j}^{CM}={\left(\frac{d}{3}\right)}^{3}\frac{\varepsilon_{j}-1}{\varepsilon_{j}+2}.$$

For each calculation, we used an interdipole spacing *d* << *λ*, as required for the validity of the expression for *p_j_* developed by Draine and Goodman [34,36,39]. In all cases *N* was comprised between 10^4^ and 10^5^, as required to minimize computational errors on the absolute value of the extinction cross-section and to allow reliable comparison between calculated optical properties [34,35,36,39,40]. The targets were generated ad hoc, each with a given optical constant according to whether they are latex or metal, and disposed according to the geometry of the object under consideration. In fact, target optical constants are introduced directly from experimental data into the calculation as input numerical parameters, independently of composition or geometry, and without any need for the interpolation with analytical models of the optical constants [34,35,36,39,40]. Therefore, optical constant of gold [41], silver [42], and polystyrene (PS) latex beads [43] were adopted. The refractive index of the matrix surrounding the NPs was that of water at 25 °C [44]. 

Besides, the optical constants of Ag and Au NPs were corrected for the intrinsic size effects according to what described by Kreibig [16,17,40,45]. The intrinsic size effect is due to the conduction electrons mean free path being comparable to particles size *l* [17,45]. In the reasonable assumption that only the free electron behaviour is affected by the size *l* of nanoparticles, the metal optical constant can be expressed in the following way [17,45]:

(7) $$\varepsilon \left(\omega ,l\right)=\varepsilon_{\infty }\left(\omega \right)+\left[\omega_{p}^{2}\left(\frac{1}{\omega^{2}+\Gamma_{\infty }^{2}}-\frac{1}{\omega^{2}+\Gamma^{2}\left(l\right)}\right)\right]+i\left[\frac{\omega_{p}^{2}}{\omega }\left(\frac{\Gamma \left(l\right)}{\omega^{2}+\Gamma_{\infty }^{2}}-\frac{\Gamma_{\infty }}{\omega^{2}+\Gamma_{\infty }^{2}}\right)\right]$$

where $\varepsilon_{\infty }\left(\omega \right)$ is the bulk value of the optical constant at frequency *ω*, $\Gamma_{\infty }$ is the electrons relaxation frequency of the bulk metal, and *Γ(l)* is the *l*-dependent value given by the following “size equation” [17,45,46]:

(8) $$\Gamma \left(l\right)=\Gamma_{\infty }+A\frac{v_{F}}{l}$$

with *v_F_* the Fermi speed and *A* an empirical parameter usually set equal to 1 [17,45]. 

All the calculated *C_ext_* resulted from the arithmetic average over 2 orthogonal polarization directions and 27 sets of Euler angles of rotation of the target with respect to the incident plane wave (i.e., a total of 54 different orientations for each extinction cross-section plotted) to simulate the dispersion of oligomers with random orientation in a liquid solution.

The same spectral range was set in all calculations and related graphs between 300 and 900 nm to allow the comparison of the spectral behaviours of the different nanosystems modelled. 

## 3. Results

### 3.1. Homo-Aggregates of Latex Nanospheres

**Figure 1:**
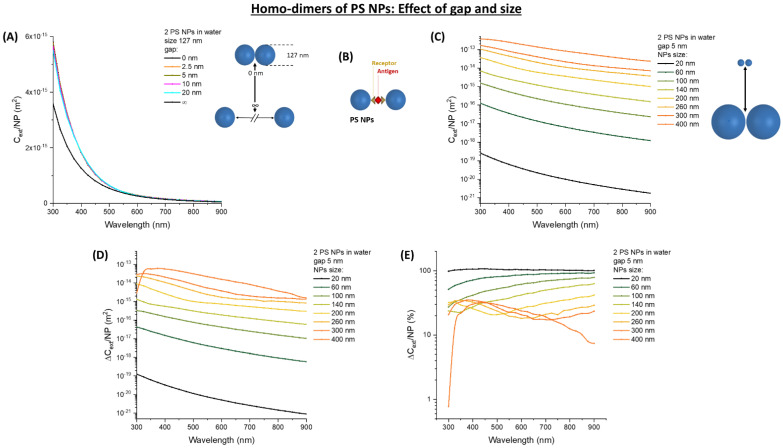
Homo-dimers of PS NPs: Effect of gap and size. (**A**) Dimer of 127 nm PS NPs in water at variable gap. (**B**) Sketch of dimer formation by antibody–antigen–antibody sandwich immunoagglutination. (**C**) *C_ext_/NP* for dimers of PS NPs with variable size. Absolute (**D**) and relative (**E**) ∆*C_ext_/NP* for dimers of PS NPs with variable size are also reported.

In order to model the optical extinction properties of oligomers of nanospheres, we started our calculations with the case of two latex spheres with a diameter of 127 nm in water. The size is intermediate between “large” (>200 nm) and “small” (<100 nm) latex spheres used in most research investigations or bioanalytical kits [1,2,24,47,48]. The gap between the two spheres is changed from infinite to 0, to show the effect of interparticle distance on *C_ext_* (Figure 1A). Clustering of the NPs is associated with an increase of the *C_ext_* per single nanoparticle (*C_ext_/NP*), which is especially pronounced below 500 nm. This change is scarcely influenced by the interparticle gap when it is varied between 20 and 0 nm. Antibodies used for surface functionalization of latex spheres and most protein antigens usually have a size of 1–3 nm [49], hence at a first approximation we can assume that interparticle distance due to the antibody–antigen–antibody sandwich (Figure 1B) is of the order of 5 nm. Keeping this gap of 5 nm, the effect of nanosphere size is shown in Figure 1C. As it is well-known from the theory of electromagnetic scattering in dielectric spheres, the extinction per single nanoparticle dramatically scales with size [10,11]. This is confirmed by the change in extinction per single nanoparticle (∆*C_ext_/NP*) passing from the monomeric to the dimeric state, as shown in Figure 1D. However, it is worth stressing that the increase in aggregate size due to dimerization is responsible for a larger relative change of the extinction, compared to the monomers, as shown in the plot of ∆*C_ext_/NP* in percentage (%) of the monomer cross-section (Figure 1E). In fact, the smaller nanospheres are associated with a change of the order of +100% in the whole spectral range from 300 to 900 nm, which becomes a few ten % in spheres > 100 nm. Regarding the detection limit and sensitivity at a low analyte concentration of a turbidimetric assay, however, the absolute change in extinction is more important [1,2,4,6,7]. This change should be such that the formation of a small number of dimers is enough to be measurable, that is not the case with small (<200 nm) latex nanospheres, according to the calculations shown in Figure 1D.

**Figure 2:**
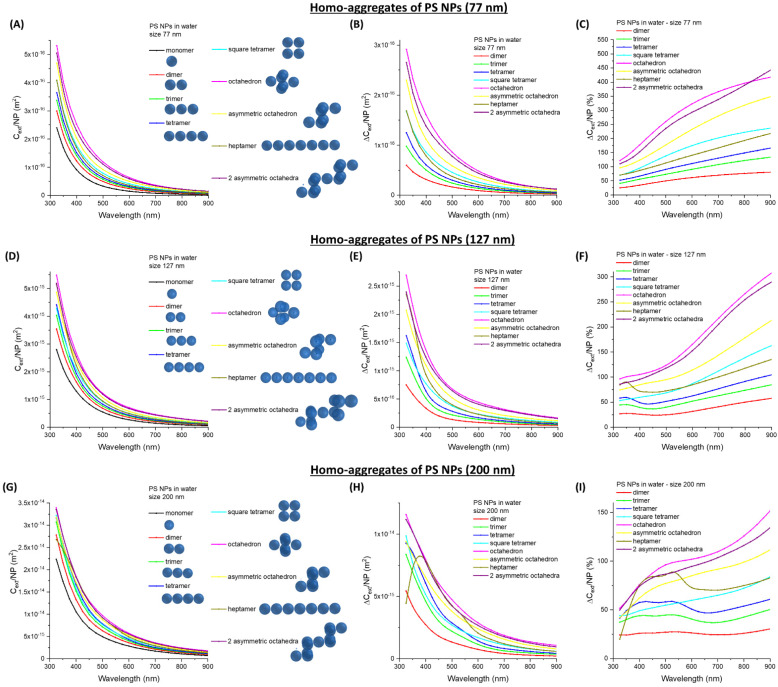
Homo-aggregates of PS NPs. (**A**) *C_ext_/NP* for aggregates of PS_77. Absolute (**B**) and relative (**C**) ∆*C_ext_/NP* are also reported. (**D**) *C_ext_/NP* for aggregates of PS_127. Absolute (**E**) and relative (**F**) ∆*C_ext_/NP* are also reported. (**G**) *C_ext_/NP* for aggregates of PS_200. Absolute (**H**) and relative (**I**) ∆*C_ext_/NP* are also reported.

Dimers are just the first type of oligomers that form at low antigen concentration [50,51,52,53]. Hence, we modelled the extinction of oligomers formed by a larger number of NPs (up to 11). Given all the possible arrangements for nanospheres in an oligomer, the calculations have been focused on some geometric arrangements that can be associated with the formation of linear, fractal, or compact clusters [50,51,52], considering three nanosphere sizes corresponding to commercially available latex beads of 77 (PS_77, Figure 2A–C), 127 (PS_127, Figure 2D–F), and 200 nm (PS_200, Figure 2G–I) [1,2,3,7]. For the three sizes considered, the trends of *C_ext_/NP*, the absolute ∆*C_ext_/NP* in m^2^, and the relative ∆*C_ext_/NP* in percentage (%) all indicate the increase of the extinction per NPs while increasing the number of spheres in the oligomer. Thus, the calculations confirm the general principle that latex clusterization is associated with the increase of extinction and with a continuously growing signal in the turbidimetric assay [1,2,4,6].

Another general trend observed from the calculations is that the more compact the oligomers are, the bigger the increase of extinction per NP, and this is especially evident for the octahedron. Nonetheless, the formation of fractal aggregates, such as the asymmetric octahedron or the two asymmetric octahedra, is more representative of immunoturbidimetric assays [50,51,53]. These types of oligomers have a larger extinction per NP compared to linear chains of spheres. The absolute ∆*C_ext_/NP* strongly depends on the wavelength, with the largest change of the order of 10^−16^ m^2^ for 77 and 127 nm NP, or 10^−15^ m^2^ for 200 nm NP, observed below 500 nm. The relative ∆*C_ext_/NP* has an opposite trend compared to the absolute changes since the percentage systematically increases with wavelength. Besides, the relative change of ∆*C_ext_/NP* is 2–4 times larger in NPs of 77 nm compared to the 200 nm ones, suggesting the rapid loss of linearity of large nanospheres when increasing the size of the clusters.

The calculations suggest that the region < 500 nm is associated with the highest sensitivity, although saturation of the turbidimeters is reached earlier than for wavelength > 500 nm, where the ∆*C_ext_/NP* is one order of magnitude smaller. Hence, the use of two wavelengths, one < 500 nm for low analyte concentrations and the other > 500 nm for normal assays, can be a simple strategy to increase sensitivity in the next generation of turbidimeters. It should be noted that performing the turbidimetric analysis in the blue portion of the electromagnetic spectrum is somehow risky due to the possible interference from several other biomolecules that can generate a nonspecific extinction signal [6,49,54]. Conversely, in single wavelength turbidimeters, the use of latex spheres requires the improvement of sensitivity beyond 500 nm [1,2,4,6,9].

### 3.2. Homo-Aggregates of Au or Ag Nanospheres

**Figure 3:**
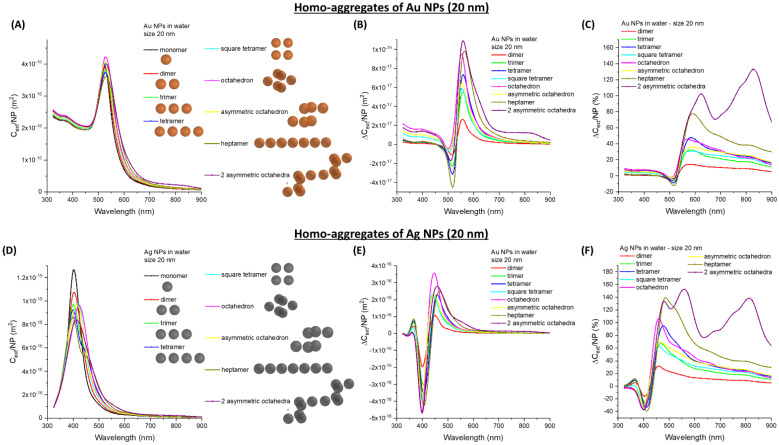
Homo-aggregates of Au or Ag NPs. (**A**) *C_ext_/NP* for aggregates of Au_20. Absolute (**B**) and relative (**C**) ∆*C_ext_/NP* are also reported. (**D**) *C_ext_/NP* for aggregates of Ag_20. Absolute (**E**) and relative (**F**) ∆*C_ext_/NP* are also reported.

The behaviour of benchmark plasmonic NPs like 20 nm Au (Au_20, Figure 3A–C) and Ag (Ag_20, Figure 3D–F) nanospheres has been investigated as a possible way to increase the sensitivity beyond 500 nm. For the sake of comparison with the previous calculations, the same geometric arrangements of latex spheres and the interparticle gap fixed at 5 nm are used. The extinction band due to the surface plasmon resonance of Au (Figure 3A) and Ag (Figure 3D) undergo a much different behaviour compared to the latex spheres. The main effect of clustering is on the plasmon band, which is red-shifted and broadened. Consequently, the ∆*C_ext_/NP* has a dispersive profile, with a negative peak for wavelengths shorter than the plasmon band of isolated Au or Ag NPs, and a positive peak at longer wavelengths. A negative value of the relative and absolute ∆*C_ext_/NP* at a specific wavelength means that the extinction cross-section per single NP (that is always a quantity ≥ 0) is lower in the aggregates than in the isolated components. The resulting dispersive trend around the maximum of the plasmon resonance is reminiscent of the differential absorbance measured in solutions of optically active compounds by circular dichroism (i.e., the Cotton effect). However, the physical origin of ∆*C_ext_/NP* in the plots of Figure 3B,C,E,F is different than in the Cotton effect because the ∆*C_ext_/NP* is obtained from the difference between the *C_ext_/NP* of the aggregated and the isolated NPs, whereas in circular dichroism the spectrum is obtained from the same solution by measuring the differential absorbance of light circularly polarized in opposite directions.

The main absolute change of *C_ext_/NP* is in the 550–650 nm range for Au NPs and 450–550 nm range for Ag NPs, instead of at shorter wavelengths as in latex NPs. These spectral ranges are coincident with those frequently exploited in turbidimeters [1,2,4,6,7]. When looking at the relative change of *C_ext_/NP*, it is also confirmed that the largest effect is located on the right side of the plasmon band. Overall, the red-shift and broadening increase with the number of NPs in the cluster, but there is also a dependence on the arrangement of NPs at parity of their number in the aggregate. This is especially observed between the symmetric and asymmetric octahedra, since the former is associated with the largest absolute and relative increase of the *C_ext_/NP*, similarly to what is found with the latex spheres.

### 3.3. Hetero-Aggregates of Metal and Latex NPs

**Figure 4:**
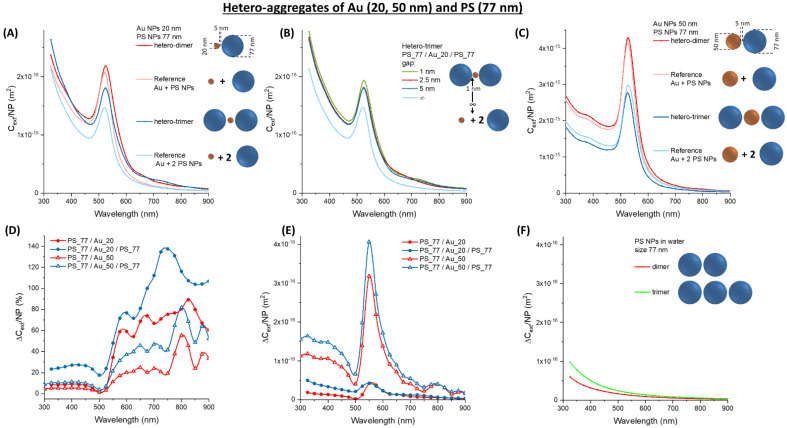
Hetero-aggregates of Au and PS NPs. (**A**) *C_ext_/NP* for aggregates of Au_20 and PS_77. (**B**) *C_ext_/NP* for a PS_77/Au_20/PS_77 trimer at various gap. (**C**) *C_ext_/NP* for aggregates of Au_50 and PS_77. The relative (**D**) and absolute (**E**) ∆*C_ext_/NP* for Au_20 or Au_50 oligomers with PS_77. (**F**) The absolute ∆*C_ext_/NP* for PS_77 homo-dimers and trimers are reported for comparison.

**Figure 5:**
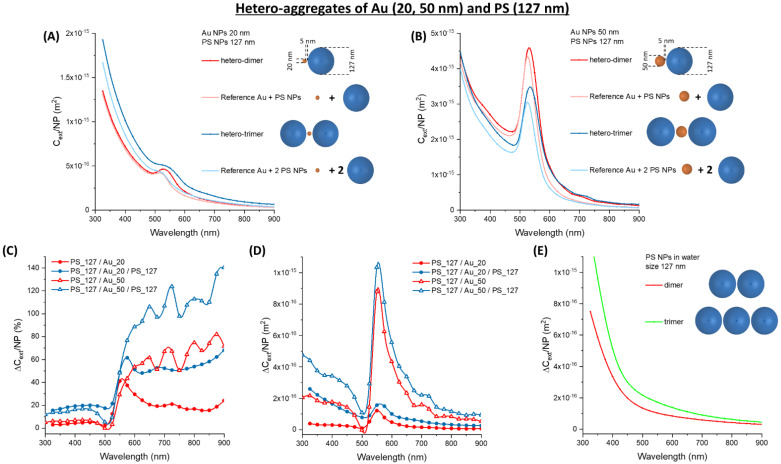
Hetero-aggregates of Au and PS NPs. (**A**) *C_ext_/NP* for aggregates of Au_20 and PS_127. (**B**) *C_ext_/NP* for aggregates of Au_50 and PS_127. The relative (**C**) and absolute (**D**) ∆*C_ext_/NP* for Au_20 or Au_50 oligomers with PS_127. (**E**) The absolute ∆*C_ext_/NP* for PS_127 homo-dimers and trimers are reported for comparison.

**Figure 6:**
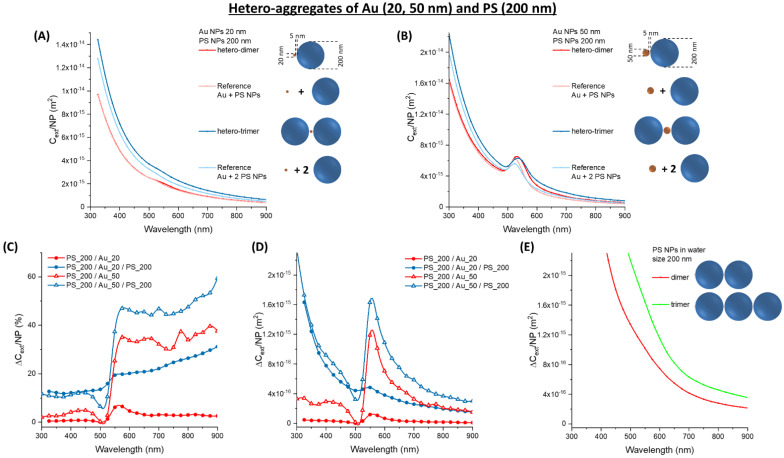
Hetero-aggregates of Au and PS NPs. (**A**) *C_ext_/NP* for aggregates of Au_20 and PS_200. (**B**) *C_ext_/NP* for aggregates of Au_50 and PS_200. The relative (**C**) and absolute (**D**) ∆*C_ext_/NP* for Au_20 or Au_50 oligomers with PS_200. (**E**) The absolute ∆*C_ext_/NP* for PS_200 homo-dimers and trimers are reported for comparison.

It should be noted that, in the region > 550 nm the absolute ∆*C_ext_/NP* of Au and Ag homo-aggregates is comparable to that of 77 nm latex NPs. Thus, the calculations considered the optical properties of hetero-aggregates of plasmonic and latex NPs, to verify the occurrence of a synergistic effect leading to an improvement of the optical extinction upon clusterization. The calculations focused on dimers and trimers of Au and latex NPs, starting with 20 nm (Au_20) or 50 nm (Au_50) metal NPs and 77 nm latex (PS_77) NPs, subsequently considering 127 nm (PS_127) and 200 nm (PS_200) latex NPs (Figure 4, Figure 5 and Figure 6). The *C_ext_/NP* for the oligomers was compared in all cases with that of the corresponding isolated NPs. In the Au_20/PS_77 and PS_77/Au_20/PS_77 oligomers (Figure 4A), a remarkable increase of *C_ext_/NP* is observed (larger for the trimer), as well as a red-shift of the surface plasmon band. The modification of the optical properties shows little dependence on the interparticle gap, when it is varied between 5 and 1 nm (Figure 4B), which are the values typically measured by transmission electron microscopy for immuno-aggregates of metal NPs [24,55].

In the Au_50/PS_77 and PS_77/Au_50/PS_77 oligomers (Figure 4C), we still observe the red-shift and increase of *C_ext_/NP*, although the change is less evident than in the Au_20 case. This is very appreciable in the plot of the relative ∆*C_ext_/NP* (Figure 4D), where the percent change of the Au_20 oligomers is almost twice that of the Au_50 ones. Contrary to pure latex NPs, the optical properties of the hetero-aggregates mostly change in the spectral region > 500 nm, as desirable for typical turbidimeters. Furthermore, the change on the left side of the plasmon band is weakly negative in the hetero-aggregates, showing an edge in the differential cross-section instead of the dispersive trend of pure Au NPs. In the 300–450 nm range, the interband transitions of Au NPs overlap with the scattering profile of the latex spheres. The two contributions are no more discernible in the Au_20 oligomers, while the interband transitions of gold prevail in the Au_50 oligomers.

As stated before, what is more important for turbidimetric assays is the absolute change of extinction, ∆*C_ext_/NP* (Figure 4E), which is one order of magnitude larger for the Au_50 oligomers compared to the Au_20 ones. This is due to the large cross-section of 50 nm gold NPs, which dominates the optical extinction spectrum. For both the Au_50 and Au_20 oligomers, the change is maximum on the right side of the plasmon band, due to its broadening and red-shift. This change is located exactly in the 500–600 nm range used in most turbidimeters. To better stress the advantage brought from hetero-aggregates, the ∆*C_ext_/NP* for PS_77 dimers and trimers are also reported (Figure 4F). One can see that ∆*C_ext_/NP* in the 500–600 nm range is of the order of 10^−17^ m^2^ for the Au_20 hetero-aggregates and the PS_77 homo-aggregates, while it exceeds 10^−16^ m^2^ for the Au_50 hetero-aggregates. This indicates a clear advantage in the sensitivity of the turbidimetric assay when a combination of Au_50 and PS_77 is used instead of pure latex NPs.

Then, the calculations considered Au_20 and Au_50 hetero-aggregates with latex NPs of 127 nm (PS_127). In the Au_20 hetero-aggregates (Figure 5A), the optical extinction is dominated by their scattering profile, due to the large size of the latex NPs. Nonetheless, the red-shift and broadening of the surface plasmon band in the hetero-dimers and trimers are still appreciable. In the Au_50 case, the extinction profiles are dominated by the surface plasmon band (Figure 5B), which makes noticable the red-shift and band broadening in the oligomers. The increased scattering contribution from the PS_127 explains why the relative ∆*C_ext_/NP* (Figure 5C) is of the same order of magnitude (ca. 50% in the 500–600 nm range) for the Au_20 and the Au_50 oligomers. This is not the case of the absolute ∆*C_ext_/NP* (Figure 5D), which in the 500–600 nm range is of the order of 10^−16^ m^2^ for the Au_20 hetero-aggregates, while reaching 10^−15^ m^2^ for the Au_50 ones. As a comparison, the PS_127 homo-aggregates have a ∆*C_ext_/NP* of 10^−16^ m^2^ in the same spectral range (Figure 5E). Importantly, the ∆*C_ext_/NP* of Au_50 hetero-aggregates in the 500–600 nm range is comparable to that of the PS_127 homo-aggregates in the blue region of the spectrum. Hence, at low analyte concentration, a mixture of Au_50 and PS_127 can improve the sensitivity of the turbidimetric assay of one order of magnitude compared to PS_127 alone. This is possible without resorting to the double-wavelength readout, which is more expensive and complex to be implemented in analytical routines and subjected to nonspecific signals in the blue spectral region.

When Au_20 and Au_50 hetero-aggregates with latex NPs of 200 nm (PS_200) are considered, the scenario is different from the previous cases due to the prevalence of scattering contribution from the dielectric spheres. The surface plasmon resonance in the Au_20 oligomers is barely detectable (Figure 6A), while the Au_50 oligomers still have a well-defined plasmon band (Figure 6B). Even in the 300–450 nm range, the interband transitions of Au NPs overlap with the scattering profile of the latex spheres and the two contributions are no more discernible from each other.

The effect of clustering remains that of producing a red-shift and broadening of the plasmon band, which is better appreciated from the plot of the relative ∆*C_ext_/NP* (Figure 6C). Indeed, the edge in concomitance of the plasmon resonance is neat only for the Au_50 oligomers. In fact, the plots of absolute ∆*C_ext_/NP* for the Au_20 hetero-aggregate (Figure 6D) resemble that of latex homo-aggregates (Figure 6E), but with extinction values that are one order of magnitude lower. Only the Au_50 hetero-aggregates have comparable absolute ∆*C_ext_/NP* than the PS_200 homo-aggregates, in the 500–600 nm range.

Although the above-mentioned differences due to the size of the latex spheres, the relative and absolute ∆*C_ext_/NP* always show a dispersive trend around the maximum of the plasmon resonance of the isolated gold nanoparticles. The spectral position of the plasmon resonance and the related centre of the dispersive trend in ∆*C_ext_/NP* depend on the size and shape of the isolated Au NPs, hence can be changed by acting on these parameters.

Overall, there are no apparent advantages in using hetero-aggregates of Au and 200 nm latex NPs but, as stated previously, the PS_200 NPs are not the best choice for turbidimetric assays due to rapid saturation of the signal at large analyte concentration.

**Figure 7:**
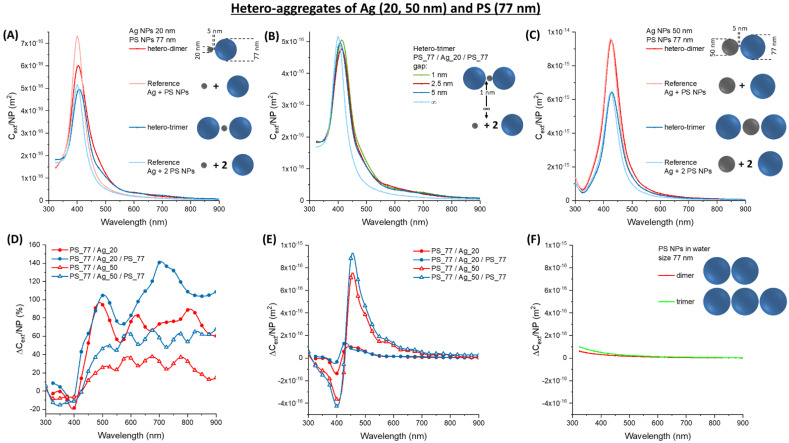
Hetero-aggregates of Ag and PS NPs. (**A**) *C_ext_/NP* for aggregates of Ag_20 and PS_77. (**B**) *C_ext_/NP* for a PS_77/Ag_20/PS_77 trimer at various gap. (**C**) *C_ext_/NP* for aggregates of Ag_50 and PS_77. The relative (**D**) and absolute (**E**) ∆*C_ext_/NP* for Ag_20 or Ag_50 oligomers with PS_77. (**F**) The absolute ∆*C_ext_/NP* for PS_77 homo-dimers and trimers are reported for comparison.

**Figure 8:**
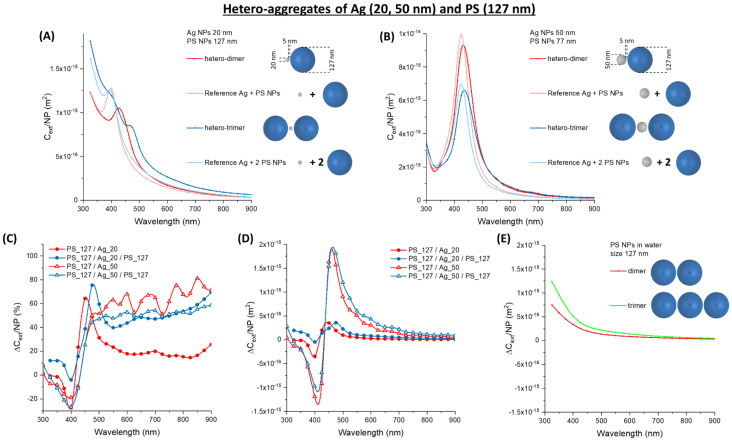
Hetero-aggregates of Ag and PS NPs. (**A**) *C_ext_/NP* for aggregates of Ag_20 and PS_127. (**B**) *C_ext_/NP* for aggregates of Ag_50 and PS_127. The relative (**C**) and absolute (**D**) ∆*C_ext_/NP* for Ag_20 or Ag_50 oligomers with PS_127. (**E**) The absolute ∆*C_ext_/NP* for PS_127 homo-dimers and trimers are reported for comparison.

**Figure 9:**
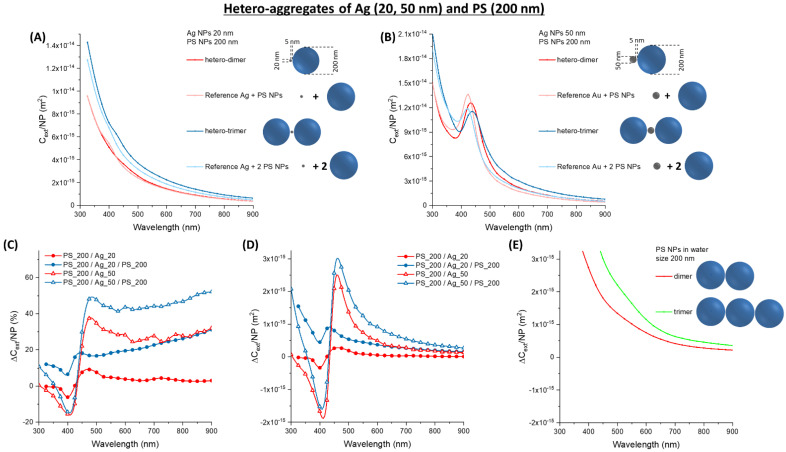
Hetero-aggregates of Ag and PS NPs. (**A**) *C_ext_/NP* for aggregates of Ag_20 and PS_200. (**B**) *C_ext_/NP* for aggregates of Ag_50 and PS_200. The relative (**C**) and absolute (**D**) ∆*C_ext_/NP* for Ag_20 or Ag_50 oligomers with PS_200. (**E**) The absolute ∆*C_ext_/NP* for PS_200 homo-dimers and trimers are reported for comparison.

While Au NPs are the standard nanomaterials for optical or colorimetric sensing due to their long-term chemical stability [15,17,18], Ag NPs are renowned for the best plasmonic performance in the visible range [16]. For instance, the extinction cross-section of Ag NPs is 5–10 times larger than Au NPs with the same morphology, which may suggest a proportionally greater effect in hetero-aggregates [16,17,40]. Therefore, the calculations were performed also on dimers and trimers containing 20 nm (Ag_20) or 50 nm (Ag_50) silver NPs and 77 nm (PS_77), 127 nm (PS_127), or 200 nm (PS_200) latex NPs (Figure 7, Figure 8 and Figure 9). The surface plasmon band of Ag_20 hetero-aggregates is sensibly lower than in isolated NPs (Figure 7A), and the red-shift and broadening are observed also in this case like in the oligomers with Au and latex NPs.

In the PS_77/Ag_20/PS_77 trimer, the red-shift depends on the interparticle gap in the 1–5 nm range (Figure 7B), being the maximum for the smallest gap. However, band broadening and increase of optical density in the red spectral window is scarcely affected by the gap size.

In Ag_50 oligomers (Figure 7C), the change in the plasmon resonance band is less evident. However, the relative ∆*C_ext_/NP* (Figure 7D) is of comparable entity for the Ag_20 and the Ag_50 hetero-aggregates and exceeds 40% for wavelengths > 450 nm. The absolute ∆*C_ext_/NP* (Figure 7E) shows the maxima in the 450–500 nm range, which is not the preferred one for ordinary turbidimetry. The change in *C_ext_/NP* is of the order of 10^−17^ m^2^ for Ag_20 and 10^−16^ m^2^ for Ag_50, i.e., comparable or one order of magnitude larger than in PS_77 homo-aggregates (Figure 7F).

The coupling of Ag NPs with 127 nm latex nanospheres has a more evident effect on plasmon resonance. In Ag_20 (Figure 8A) and Ag_50 (Figure 8B), the red-shift and broadening of the plasmon band is very evident and corresponds to an increment of the optical density in the spectral region >450 nm, as shown by the plot of the relative ∆*C_ext_/NP* (Figure 8C). This increment is accompanied by a negative dip at 400 nm, where the plasmon resonance of isolated Ag NPs is peaked. The absolute change of *C_ext_/NP* (Figure 8D) is twice that of the hetero-aggregates with PS_77, but it is still located prevalently in the 450–550 nm range. Compared to homo-aggregates of PS_127 (Figure 8E), the ∆*C_ext_/NP* of hetero-aggregates with Ag_50 is more than one order of magnitude larger in the 450–550 nm range.

The extinction cross-section of oligomers of Ag_20 and 200 nm latex nanospheres is dominated by the scattering profile of the big dielectric spheres (Figure 9A), while the surface plasmon resonance is easily detectable only in the Ag_50 oligomers (Figure 9B). The red-shift and broadening of the plasmon band are found also in this case, although with a relative ∆*C_ext_/NP* (Figure 9C) lower than in the oligomers with PS_77 and PS_127. This is the expected consequence of the larger contribution from the 200 nm latex nanospheres, that undergo a small relative change of optical density passing from the isolated to the hetero-aggregate configuration. The absolute change of *C_ext_/NP* (Figure 9D) is comparable to that of the oligomers with PS_77 and PS_127, as well as to the homo-aggregates of PS_200 (Figure 9E) in the 450–550 nm range.

### 3.4. Optimising Hetero-Aggregates of Metal and Latex NPs

**Figure 10:**
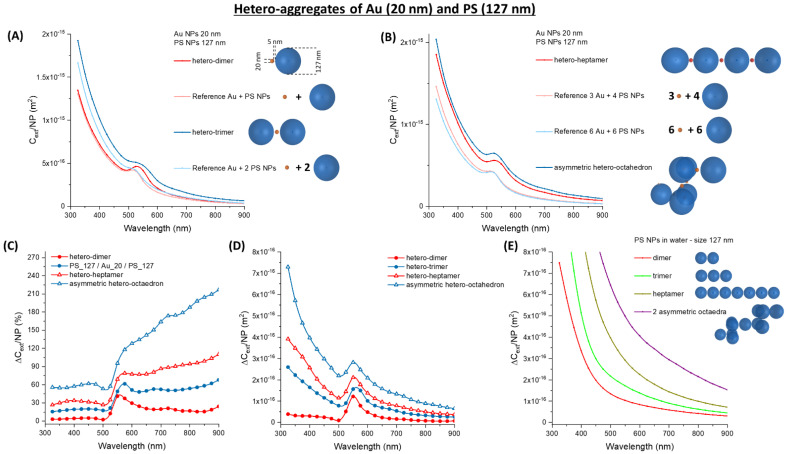
Hetero-aggregates of Au and PS NPs. (**A**) *C_ext_/NP* for a dimer and a trimer of Au_20 and PS_127. (**B**) *C_ext_/NP* for a heptamer and an asymmetric octahedron of Au_20 and PS_127. The relative (**C**) and absolute (**D**) ∆*C_ext_/NP* for Au_20 oligomers with PS_127. (**E**) The absolute ∆*C_ext_/NP* for PS_127 homo-aggregates are reported for comparison.

The calculations on plasmonic-dielectric hetero-aggregates identified gold NPs with the latex spheres of 77 or 127 nm as the optimal combination for immuno-turbidimetric assays. These combinations are featured by a remarkable change of the optical density in the preferred range for turbidimetry (550–600 nm), which is larger than in the corresponding homo-aggregates of latex spheres. Particles size is lower than the 200 nm threshold, for which a limited dynamic range and linearity interval has been reported [1,2]. Nonetheless, the size of Au and latex spheres has to be selected carefully, to obtain a real advantage compared to latex alone. This is further demonstrated by a series of calculations of *C_ext_/NP* in hetero-aggregates composed of 7 or 12 particles arranged, respectively, linearly or in asymmetric octahedra (Figure 10).

Calculations considered the two opposite combinations of small gold spheres (Au_20) with the PS_127 NPs or the large gold particles (Au_50) with the smallest latex beads (PS_77). When Au_20 and PS_127 are combined, the *C_ext_/NP* continuously grows with the number of particles in the aggregate, going from the dimer to the trimer (Figure 10A), the heptamer and the asymmetric octahedron (Figure 10B). According to the plot of the relative ∆*C_ext_*/*NP* (Figure 10C), the increment is always at wavelengths > 550 nm, although it is not associated with a dramatic change of the shape of the plasmon band. This result agrees with the previous observations of red agglomerates between 3 μm latex and 13 nm Au NPs [56].

The absolute change of ∆*C_ext_/NP* is of the order of 10^−16^ m^2^ (Figure 10D), which is comparable to the absolute ∆*C_ext_/NP* of homo-aggregates (Figure 10E). This means that there is no advantage in combining Au_20 with PS_127.

**Figure 11:**
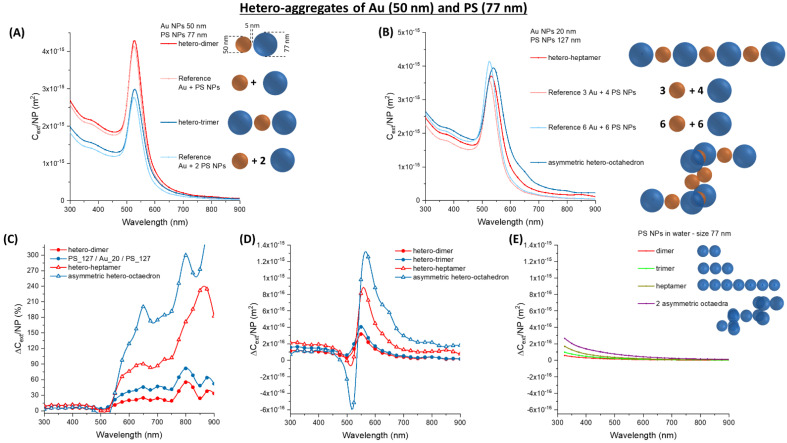
Hetero-aggregates of Au and PS NPs. (**A**) *C_ext_/NP* for a dimer and a trimer of Au_50 and PS_77. (**B**) *C_ext_/NP* for a heptamer and an asymmetric octahedron of Au_50 and PS_77. The relative (**C**) and absolute (**D**) ∆*C_ext_/NP* for Au_50 oligomers with PS_77. (**E**) The absolute ∆*C_ext_/NP* for PS_77 homo-aggregates are reported for comparison.

When Au_50 NPs are combined with PS_77 spheres, the change in *C_ext_/NP* also has a continuously growing trend passing from the dimer to the trimer (Figure 11A), the heptamer and the asymmetric octahedron (Figure 11B). The relative increment of ∆*C_ext_/NP* is located at wavelengths > 550 nm as well (Figure 11C). However, this change is of the order of 10^−15^ m^2^ in the absolute ∆*C_ext_/NP* (Figure 11D), which is more than one order of magnitude larger than in homo-aggregates (Figure 11E). Hence, the combination of Au_50 and PS_77 NPs provides an advantage of sensitivity compared to the separated components.

**Figure 12:**
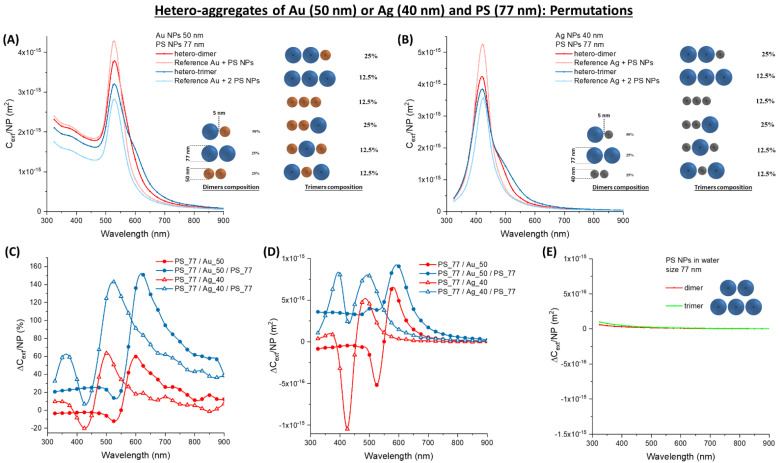
Permutations in hetero-aggregates of Au or Ag and PS NPs. (**A**) Permutation-weighted *C_ext_/NP* for a dimer and a trimer of Au_50 and PS_77. (**B**) Permutation-weighted *C_ext_/NP* for a dimer and a trimer of Ag_40 and PS_77. The relative (**C**) and absolute (**D**) ∆*C_ext_/NP* for Au_20 oligomers with PS_127. (**E**) The absolute ∆*C_ext_/NP* for PS_77 homo-aggregates are reported for comparison.

A more accurate prediction of the optical properties of a 1:1 mixture of Au_50 and PS_77 NPs can be achieved by accounting for the permutations of gold and latex NPs in the aggregates. For instance, in the case of the simplest immuno-aggregate occurring at the lowest antigen concentration, the dimer, these permutations are Au_50/PS_77 (50%), PS_77/PS_77 (25%) and Au_50/Au_50 (25%) (Figure 12A). The resulting *C_ext_/NP* weighted on all the possible permutations still shows a remarkable broadening of the main extinction band (Figure 12A). This is reasonable considering the additional contribution due to the modification of the surface plasmon band in the 25% of Au_50/Au_50 homodimers. The permutation-weighted *C_ext_/NP* was calculated also for a 1:1 mixture of Ag_40 and PS_77 NPs. Thus, the average is performed on the Ag_40/PS_77 (50%), PS_77/PS_77 (25%) and Ag_40/Ag_40 (25%) permutations. The resulting *C_ext_/NP* retains the red-shift and broadening of the plasmon band, especially in the 450–550 nm range as in the previous calculations with silver particles.

When trimers with a linear arrangement are considered, the number of permutations is 6 (Figure 12A,B). The permutation-weighted *C_ext_/NP* or Au_50 and Ag_40 NPs with PS_77 also show a remarkable broadening of the plasmon band. The relative ∆*C_ext_/NP* (Figure 12C) is located again in the 550–650 nm range for the Au trimers. For Ag trimers, ∆*C_ext_/NP* extends from 450 to 600 nm, which is a larger interval than in dimers. The absolute ∆*C_ext_/NP* (Figure 12D) is of the order of 10^−15^ m^2^ in both cases, a higher value than in PS_77 homo-aggregates.

**Figure 13:**
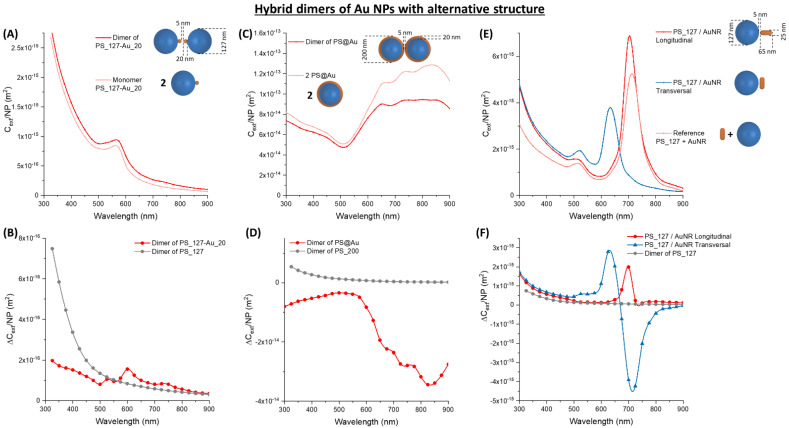
Hybrid dimers of other Au nanostructures. (**A**) *C_ext_/NP* for a dimer of PS_127-Au_20. (**B**) The absolute ∆*C_ext_/NP* for the PS_127-Au_20 and the PS_127 dimers. (**C**) *C_ext_/NP* for a dimer of PS@Au core-shell (core diameter 160 nm, shell thickness 20 nm). (**D**) The absolute ∆*C_ext_/NP* for the PS@Au core@shell and the PS_200 dimers. (**E**) *C_ext_/NP* for a dimer of PS_127 and a Au NR (cylinder with hemispherical caps, long 65 nm, diameter 25 nm) with longitudinal (L) or transversal (T) orientation. (**F**) The absolute ∆*C_ext_/NP* for the AuNR/PS_127 L and T dimers and the PS_127 dimers.

The Au_50, Ag_40, and PS_77 NPs are easily implementable in immunoturbidimetric assays because they are all commercially available at a relatively low cost and have well-known surface chemistry for immobilization of antibodies or other targeting groups that specifically bind analytes in sandwich configurations [1,2,18,24,57]. On the other hand, the plasmonic response dramatically changes with the shape of the metal object [16,17,27,28,58], hence additional calculations were performed on more complex metal nanostructures, such as latex beads decorated with gold NPs and PS@Au core@shell whose synthesis has been reported in the literature [27,59], or commercially available gold nanorods [60] (Figure 13).

In the case of a hybrid bead made of a gold nanoparticle (20 nm) attached on top of a latex particle (127 nm), a remarkable change of the extinction cross-section is observed already in the monomer (Figure 13A), due to the tight coupling between the metal and the dielectric moieties. The extinction of the isolated hybrid bead is featured by a plasmon resonance band shifted beyond 550 nm and over imposed to the scattering profile of the latex sphere. After dimer formation, with a gap of 5 nm between the Au_20 NPs, there is an almost homogeneous increase of the optical density in the whole spectral range. This can be interpreted as the combined effect of the increase of light scattering in the blue portion of the spectrum due to the proximity of the two latex spheres, and the increase of extinction in the red portion of the spectrum due to the coupling of the two Au NPs. In fact, the ∆*C_ext_/NP* is lower than that of the PS_127 dimer for wavelength < 550 nm (Figure 13B), due to the larger separation between the latex spheres in the hybrid dimer. Beyond 550 nm, the ∆*C_ext_/NP* is slightly higher than in the PS_127 dimer, thanks to the plasmonic coupling of the Au_20 NPs. However, a change of less than one order of magnitude is not enough to justify the synthetic complexity of the PS_127-Au_20 hybrid.

When a PS@Au core@shell with 20 nm thick gold shell and latex core with 160 nm of diameter is considered, the *C_ext_/NP* of the dimer is homogeneously lower in the whole spectral range (Figure 13C). The cores@shells with dielectric core and gold shell are renowned for their broad plasmonic band in the red portion of the visible spectrum, as happens in the PS@Au case. This extinction band is less intense in the dimer because of the red-shift of the plasmon resonance of the dimer in the near-infrared, due to the strong plasmonic coupling between the two nanospheres. The change of extinction due to this red-shift is so intense that overwhelms the increase of the scattering contribution due to the formation of the dimer. The resulting absolute ∆*C_ext_/NP* is more than one order of magnitude lower than the PS_200 NPs (Figure 13D).

Finally, the effect of dimerization on a cylindrical AuNR with hemispherical caps (65 nm total length and diameter of 25 nm) with PS_127 was investigated (Figure 13E). Nanorods of gold are known to exhibit two plasmon bands, one most intense at longer wavelengths, that corresponds to the polarization of conduction electrons along the main rod axis, and a second resonance, less intense, located at a similar wavelength as in nanospheres, due to polarization along the short axis [16,17,40,61]. The spectral location of the main resonance is tuneable by changing the aspect ratio of the rod, i.e., the ratio between the main and the minor axis [16,17,40,61,62]. It should be noted that asymmetric NPs can bind the latex moiety with a different orientation and, for the AuNR, we considered the two limiting cases of longitudinal or transversal binding along the dimer axis. The orientation makes a big difference in the extinction of the hetero-dimer, where the plasmon resonance band is blue-shifted from 720 to 700 nm for the longitudinal configuration, or 620 nm for transversal orientation. For transversal orientation, the large blue-shift originates a dispersive profile of the ∆*C_ext_/NP* curve. Nevertheless, the resulting maxima of ∆*C_ext_/NP* are in a different spectral region depending on the orientation (Figure 13F), which makes difficult a prediction on the behaviour in real systems, where the orientation of the nanorod usually cannot be predetermined. Nonetheless, the ∆*C_ext_/NP* can be more than one order of magnitude larger than in the PS_127 dimer in the 600–650 or 700–750 nm range. Although this range is not the preferred one for turbidimetry, the resonance of nanorods can be shifted by changing their aspect ratio, to seek an optimal spectral position.

## 4. Discussion

**Figure 14:**
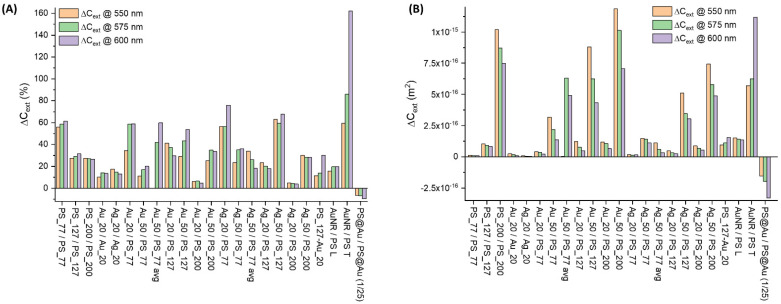
Comparative results for dimers. Relative (**A**) and absolute (**B**) ∆*C_ext_/NP* for the homo- and hetero-dimers of this study, computed at 550, 575, and 600 nm.

Ideally, the extinction properties of a colloidal solution for immunoturbidimetric assays should undergo a high change at low analyte concentration, when oligomers like dimers, trimers, etc. are formed, followed by a continuous increase of the signal at higher analyte concentration, over the whole range of analytical interest [1,2,4,6,7,14]. Importantly, this should come without saturation of the signal beyond the dynamic range of the turbidimeter and without losing the linearity in the upper limit of analyte concentration [1,2,4,6,7,14]. Although latex beads have excellent performances and have been used for several decades for immunoturbidimetric detection, their extinction properties do not allow for meeting all the features of the ideal reagent for immunoturbidimetric assays of analytes that must be measured over several orders of magnitude of concentration [6,9]. On the other hand, the responsivity of the surface plasmon resonance in noble metal NPs (Au, Ag) upon changes of the dielectric environment, has been exploited multiple times for optical sensing, even for ultrasensitive assays [14,15,18,19]. Hence, the optical properties of oligomers containing both latex and noble metal NPs have been studied to identify possible appropriate combinations with positive prospects in immunoturbidimetry. The results on the relative ∆*C_ext_/NP* in homo- and hetero-dimers are summarized in Figure 14A. The largest values (50–60% at 550, 575, or 600 nm) are reached when metal and latex NPs are coupled together. Exceptions are the PS_77 homo-dimer, which undergoes a similar change, and hetero-dimers with PS_200, which have a change of ca. 10% due to the dominant contribution of the dielectric particle over the plasmonic one.

More complex nanostructures like the PS_127-Au_20 or the PS@Au core@shell do not provide appreciable advantages over the hetero-aggregates of simple spheres. The AuNR may be a convenient choice if the binding with latex particles happens with a transversal geometry that is associated with the largest relative increase of ∆*C_ext_/NP*. However, the behaviour of AuNR in larger hetero-oligomers deserves a detailed and specific study to verify their compatibility with a monotonic increase of the optical extinction at a fixed wavelength, as required in ordinary turbidimetry.

In any case, the absolute ∆*C_ext_/NP*, summarized in Figure 14B, is the relevant parameter to predict the sensitivity of a real immunoturbidimetric assay. In this case, the size of the NPs dominates the histogram, i.e., the particles with a size of 200 nm have the largest ∆*C_ext_/NP*. Remarkably, hetero-dimers of Au_50 with PS_77 and PS_127 have comparable performances with dimers containing PS_200 NPs. Ag NPs perform slightly less than Au analogues in the 550–600 nm range because the plasmon resonance of silver nanospheres is blue-shifted more than 100 nm from that of gold ones [16,17,63]. Consequently, a larger ∆*C_ext_/NP* is expected in the 450–500 nm range for Ag hetero-aggregates. More complex plasmonic NPs have a heterogeneous response, suggesting that the cost in the realization of complex architectures differing from simple metal nanospheres is justified only after a specific effort of optimization of the ∆*C_ext_/NP*, which is not unequivocally satisfactory for the morphologies considered in this study.

**Figure 15:**
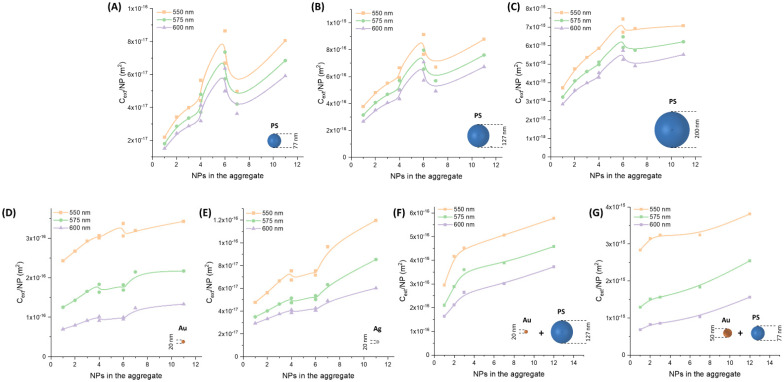
Extinction versus number (N) of particles in the aggregate at 550, 575, and 600 nm. (**A**) PS_77. (**B**) PS_127. (**C**) PS_200. (**D**) Au_20. (**E**) Ag_20. (**F**) Au_20 with PS_127. (**G**) Au_50 with PS_77.

In the plot of Figure 14B there is an obvious prevalence of ∆*C_ext_/NP* from larger particles. However, this is generally accompanied by a rapid loss of linearity while increasing the size of the oligomers. This is shown, for instance, in Figure 15, where the *C_ext_/NP* at 550, 575, and 600 nm are reported for the oligomers of PS_77 (Figure 15A), PS_127 (Figure 15B), and PS_200 (Figure 15C) as a function of the number of NPs. One can see how the increase of *C_ext_/NP* has a higher slope for smaller particles, while it reaches almost saturation beyond 8–10 NPs in the PS_200 spheres. Besides, the morphology of the cluster is more influential on the *C_ext_/NP* of the smallest latex spheres (PS_77), while being not so crucial for the PS_200.

When the Au_20 homo-aggregates are considered (Figure 15D), the dependence of *C_ext_/NP* on the number of particles also shows a saturation after 8–10 NPs at 550 nm, whereas at 600 nm there is continuous growth. The extinction cross-section per single NP is of the order of 10^−16^ m^2^, not far from that of PS_127 homo-aggregates. The different trends at 550 and 600 nm are a consequence of the progressive red-shift of the plasmon resonance in Au NPs homo-aggregates, which in oligomers is featured by an increase of the optical density in the proximity of the extinction peak of isolated nanospheres. However, for larger aggregates the plasmon band undergoes a further red-shift and broadening, instead of continuous growth in the same spectral range as it would be required for turbidimetry. This behaviour also explains the optical extinction of Ag NPs oligomers (Figure 15E), which show a more continuous growth of the optical extinction between 550 and 600 nm but on a scale that is only of the order of 10^−17^ m^2^ (same as the PS_77 homo-aggregates). In fact, the plasmon resonance of silver nanospheres is blue-shifted compared to that of Au NPs analogues [16,63,64]. Hence, the saturation of the extinction in the 550–600 nm range is reached only for aggregates containing tens of particles [65].

When the Au_20 NPs (that perform better than Ag NPs) are combined with the PS_127 particles (that represent the best compromise among latex spheres), one expects to observe high sensitivity and persistent linearity. The increase of *C_ext_/NP* (Figure 15F) for small oligomers is steep, suggesting that the two types of particles can be successfully combined to improve the performance of turbidimetric assays. Unfortunately, the steep portion of the curve is limited to 2–3 NPs in the aggregate. For more than just 3 NPs, the trend of *C_ext_/NP* undergoes a reduction of its slope and, most relevant, the overall value of the optical extinction remains in the 10^−16^ m^2^, i.e., of the same order of magnitude of PS_127 homo-aggregates.

**Figure 16:**
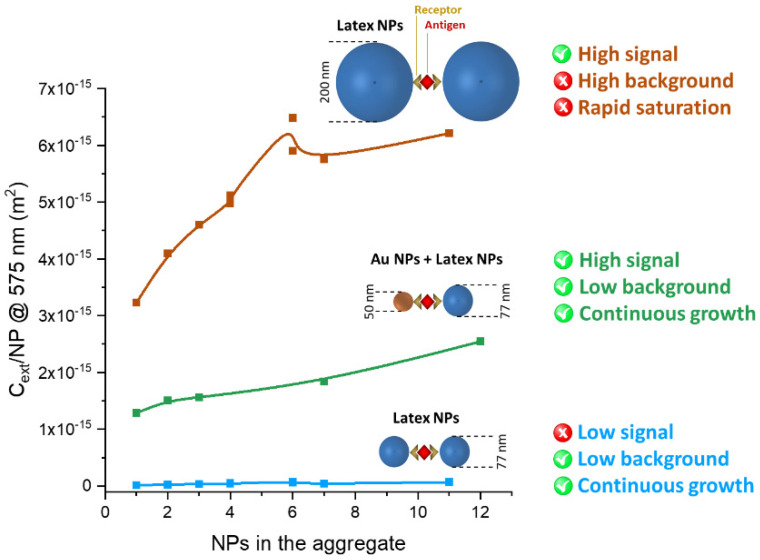
The combination of Au_50 and PS_77 NPs promises to improve the sensitivity and dynamic range in immuno-turbidimetric assays, while avoiding the low signal of PS_77 NPs homo-aggregates and the high background and limited dynamic range of large (>200 nm) latex nanospheres.

The evolution of the optical extinction is much improved when hetero-aggregates of larger gold particles (Au_50) and smaller latex spheres (PS_77) are used (Figure 15G). The *C_ext_/NP* enters the 10^−15^ m^2^ range, i.e., the same as the PS_200 homo-aggregates, but with a continuous growth from 2 to 12 NPs, instead of showing a plateau as in the 200 nm latex spheres. Hence, the combination of Au_50 plasmonic NPs and PS_77 latex particles promises to improve sensitivity and dynamic range in immuno-turbidimetric assays (Figure 16).

## 5. Conclusions

We presented a systematic study aimed at identifying a combination of plasmonic and latex NPs that can improve the detection limit of immune-turbidimetric assays, while keeping a wide dynamic range. Our calculations suggest that the combination of Au NPs with latex spheres permits a remarkable change of optical density in the preferred range for turbidimetry (550–600 nm), by the formation of oligomeric plasmonic-dielectric hetero-aggregates. The change of optical density is sensibly larger than in the corresponding homo-aggregates of latex spheres. Therefore, a mixture of plasmonic and latex NPs can meet the ideal requirement of high increment of extinction at low analyte concentration followed by a continuous increase of the optical density at higher analyte concentration, over the whole range of analytical interest. To this end, the size of Au and latex spheres must be selected carefully, to obtain a real advantage compared to latex alone. More specifically, we found an optimal response with 50 nm Au NPs and 77 nm latex spheres. The 50 nm Au NPs provide an intense variation of the optical properties already after the formation of dimers or trimers, that are the aggregates appearing first at low analyte concentration. Furthermore, the use of relatively small 77 nm latex particles is crucial to prevent the saturation of the signal read by the turbidimeter while keeping linearity in the upper limit of analyte concentration. Overall, the appropriate combination of plasmonic and latex particles promises to improve the detection limit and dynamic range of immuno-turbidimetric assays.

## Data Availability

The data presented in this study are available on request from the corresponding author.
